# Downregulation of SIRT3 Aggravates Lung Ischemia Reperfusion Injury by Increasing Mitochondrial Fission and Oxidative Stress through HIF-1*α*-Dependent Mechanisms

**DOI:** 10.1155/2022/9041914

**Published:** 2022-09-29

**Authors:** Chunxia Liu, Shenglin Pei, Huijun Dai, Zhen Liu, Mengling Ye, Hao Liu, Xiaojing He, Siyi Wu, Yi Qin, Fei Lin

**Affiliations:** ^1^Clinical Research Center for Anesthesiology, Guangxi Medical University Cancer Hospital, Nanning 530021, Guangxi Zhuang Autonomous Region, China; ^2^Key Laboratory for Basic Science and Prevention of Perioperative Organ Dysfunction, Guangxi Medical University Cancer Hospital, Nanning 530021, Guangxi Zhuang Autonomous Region, China; ^3^Engineering Research Center for Tissue and Organ Injury and Repair Medicine, Guangxi Medical University Cancer Hospital, Nanning 530021, Guangxi Zhuang Autonomous Region, China; ^4^Department of Experimental Research, Guangxi Medical University Cancer Hospital, Nanning 530021, Guangxi Zhuang Autonomous Region, China

## Abstract

Lung ischemia-reperfusion injury (LIRI) is a severe multifaceted pathological condition that can lead to poor patient outcome where oxidative stress and the resulting inflammatory response can trigger and exacerbate tissue damage in LIRI patients. Sirtuin3 (SIRT3), a member of the sirtuin family, protects against oxidative stress-related diseases. However, it remains unclear if and how SIRT3 alleviates lung injury induced by ischemia/reperfusion (I/R). Our previous study showed that lung tissue structures were severely damaged at 6 h after lung I/R in mice, however, repair of the injured lung tissue was significant at 24 h. In this study, we found that both SIRT3 mRNA and protein levels were markedly increased at 24 h after lung I/R *in vivo*. Meanwhile, inhibition of SIRT3 aggravated lung injury and inflammation, augmented mitochondrial fission and oxidative stress and increased Hypoxia-inducible factor-1*α* (HIF-1*α*) expression *in vivo*. The results suggest that SIRT3 may be an upstream regulator of HIF-1*α* expression. Knockdown of SIRT3 resulted in excessive mitochondrial fission and increased oxidative stress *in vitro*, and we found that knocking down the expression of HIF-1*α* alleviated these changes. This suggests that the SIRT3-HIF-1*α* signaling pathway is involved in regulating mitochondrial function and oxidative stress. Furthermore, inhibition of dynamin-related protein 1 (Drp-1) by the inhibitor of mitophagy, Mdivi-1, blocked mitochondrial fission and alleviated oxidative stress *in vitro*. Taken together, our results demonstrated that downregulation of SIRT3 aggravates LIRI by increasing mitochondrial fission and oxidative stress. Activation of SIRT3 inhibits mitochondrial fission and this mechanism may serve as a new therapeutic strategy to treat LIRI.

## 1. Introduction

Lung ischemia-reperfusion injury (LIRI) is a common and severe complication that imposes a significant threat to graft and recipient survival, leading to increased morbidity and mortality among patients undergoing lung transplantation [1]. Organ ischemia and subsequent reperfusion is unavoidable in lung transplantation, which usually leads to acute, sterile inflammation after transplantation called I/R injury [2]. The pathogenesis of LIRI is complex, involving many pathophysiological processes such as oxidative stress injury, calcium overload, endoplasmic reticulum stress injury, inflammatory injury, autophagy, and apoptosis [3–5].

Oxidative stress plays a crucial role in the pathophysiologic processes of I/R injury [6]. Any mismatch between pulmonary oxygen demand and supply can lead to oxidative stress and the accumulation of high amounts of reactive oxygen species (ROS) [7]. It is worth noting that because mitochondria are the main target organs and source of ROS, mitochondrial dysfunction is recognized as a key factor contributing to I/R injury [8]. Therefore, strategies to target the sources of ROS, reduce mitochondrial oxidative stress and preserve mitochondrial function are attractive approaches to ameliorate LIRI.

Under physiological conditions, mitochondria have to maintain a dynamic balance between mitochondrial fission and fusion to regulate its morphology, number and size, which are essential for mitochondrial homeostasis [9, 10]. However, in some pathological conditions, such as acute kidney injury and diabetic nephropathy [11, 12], excessive mitochondrial fission results in increased mitochondrial fragmentation which inhibits the cellular respiratory chain, leading to cellular dysfunction and aggravating tissue damage [13, 14]. Mitochondrial fission is primarily mediated by dynamin-related protein 1 (Drp-1) [15], a member of the dynamin family of large GTPases, mostly localized in the cytoplasm [16]. Drp-1 is recruited to the outer membrane of mitochondria by a variety of adaptor proteins and then aggregates along the site of mitochondrial fission in the future [17, 18]. Active Drp-1 oligomerizes in a ring-like structure and assists the constriction of outer mitochondrial membrane to divide the mitochondria into two separate mitochondria when they contract. Mdivi-1 is a cell permeable quinazolinone originally described as a selective inhibitor of Drp-1 and reported to inhibit mitochondrial fission [15]. Wu et al. proposed that Mdivi-1 prevents Drp-1 from self-assembly into the ring-like structure, thus limiting its association with mitochondria. A different study also proposed that Mdivi-1 blocks the oligomerization of Drp-1 which is necessary for its GTPase activity [19]. Other studies have demonstrated that Mdivi-1 alleviates both myocardial [20] and cerebral I/R injuries by dampening cell apoptosis [21]. However, the relationship between mitochondrial dysfunction and oxidative stress in LIRI remains unclear.

Sirtuin3 (SIRT3) is primarily localized in the mitochondria and plays an important role in the regulation of mitochondrial function and ROS production [22]. Previous studies have demonstrated that increased SIRT3 expression protects cells against oxidative stress through isocitrate dehydrogenase 2 activation [23]. In addition, SIRT3 overexpression protected from doxorubicin-induced cardiomyopathy in mice [24]. Hypoxia-inducible factors (HIFs) are key factors that control the hypoxia-inducible pathways by regulating expression of multiple genes. Hypoxia-inducible factor-1*α* (HIF-1*α*) is a major transcription factor of oxygen homeostasis-related genes and has been described as a key regulator of hypoxia induced diseases [25]. A previous study showed that SIRT3 exerts tumor suppressive effects by suppressing ROS and regulating HIF-1*α* [26]. However, the specific roles of SIRT3 and HIF-1*α* in LIRI remain largely unclear.

Given the importance of SIRT3 in regulating mitochondrial function [27], we hypothesized that SIRT3 suppression will lead to excessive mitochondrial fission and increase oxidative stress by altering expression of HIF-1*α*. Furthermore, we speculated that Mdivi-1 could reduce mitochondrial oxidative stress after inhibiting mitochondrial fission.

## 2. Materials and Methods

### 2.1. Experimental Animals

Experiments were approved by the Institutional Animal Care and Use Committee of Guangxi Medical University Cancer Hospital, and strictly followed the Institute's guidelines for the care and use of laboratory animals. Sixty male wild-type C57BL/6 J mice (6–8 weeks, 20–22 g) were purchased from the Animal Center of Guangxi Medical University (Nanning, China) and were adapted to the laboratory environment for 2 weeks before the experiments were conducted. The mice were housed in specific pathogen-free conditions and ventilated polycarbonate cages with dimensions of 325x210x150 mm (less than 5 mice per cage, 23°C, 50% humidity, 12 h light/dark cycle) with free access to sterilized food and water.

### 2.2. Mouse Model of Lung I/R and Experimental Design

Mice were randomly divided into five groups: Sham, DMSO- (dimethyl sulfoxide-) treated, a group treated with the SIRT3 selective inhibitor 3-TYP (3-(1H-1,2,3-triazol-4-yl) pyridine) dissolved in DMSO (Selleck Chemicals, USA), I/R, and I/R+3-TYP groups (*n* = 12 per group for all experiments). The mice were anaesthetized with 50 mg/kg intraperitoneal injection of pentobarbital and then, subjected to the procedure to occlude the left pulmonary hilum with a microvascular clamp. After 60 min of ischemia, the clamp was removed to allow recovery of the blood flow for 24 h before the mice were killed by carotid artery bloodletting for the I/R and I/R+3-TYP groups, while the mice in the Sham, DMSO, and 3-TYP groups only underwent a thoracotomy, and the left lung was collected for subsequent experiments after modeling. The mice in the 3-TYP and I/R +3-TYP groups received 50 mg/kg of 3-TYP by intraperitoneal injection every 2 days for a total of three times before the surgery [28–30]. The DMSO group was given an equal volume of DMSO at the same time points as the 3-TYP-treated group.

### 2.3. Cell Culture and Hypoxia/Reoxygenation (H/R)

The RAW 264.7 murine macrophages cell line was purchased from Procell Life Science&Technology Co.,Ltd. The cultured cells were incubated at 37°C in a humidified atmosphere of 5% CO_2_ and 95% O_2_. The H/R model of cells was established *in vitro* as previously described [13, 31]. Briefly, RAW 264.7 cells were cultured in serum-fed and glucose-free medium (Gibco, USA) and exposed to hypoxic conditions (94% N_2_, 5% CO_2_, 1% O_2_) at 37°C for 1 h. Then, the medium was replaced with a glucose-containing medium and cells were allowed to grow in 5% CO_2_ and 95% O_2_ for 24 h according to the requirements of the experiment.

### 2.4. Histological Analysis

Lung tissues harvested from the different groups of C57BL/6 J mice were fixed in 4% paraformaldehyde and embedded in paraffin, sectioned at a thickness of 4 *μ*m and stained with hematoxylin and eosin (H&E) and the tissue morphology was observed. The histological changes were assessed blind using a light microscope by two independent researchers. The degree of lung injury was estimated and acute lung injury scores were evaluated as previously described [32].

### 2.5. Lung Wet-to-Dry Ratio

The lung wet/dry (W/D) ratio was measured as an indicator of pulmonary edema and congestion. As we described previously [33], the lower lobe of the left lung was immediately weighed after the mice were sacrificed, incubated at 60°C for 96 h, and then weighed again. The weights were used to calculate the wet-to-dry ratio.

### 2.6. Enzyme-Linked Immunosorbent Assays (ELISA)

The concentrations of interleukin-1*β* (IL-1*β*) and interleukin-6 (IL-6) in left lung tissue homogenate or in bronchoalveolar lavage fluid (BALF) of left lung after modeling were estimated using specific ELISA kits (Elabscience Biotechnology, Wuhan, China) for mice according to the manufacturer's instructions.

### 2.7. Transmission Electron Microscopy (TEM)

Left lung tissues from different groups were taken within 1 to 3 minutes after modeling, cut into pieces of about a millimeter, and fixed in 2.5% glutaraldehyde for at least 2 h. After embedding in resin, the samples were cut into ultrathin slices by an ultramicrotome and observed with an H-7560 transmission electron microscope (H7560, Tokyo, Japan).

### 2.8. Immunofluorescence Staining

The left lung tissues from different groups were fixed with 4% paraformaldehyde, embedded in paraffin and sectioned at a thickness of 4 *μ*m. After dewaxing and rehydration treatment, the slices were immersed in 0.01 M citrate buffer (pH 6.0) and boiled in a pressure cooker for 10 minutes. The slides were incubated with primary antibodies for SIRT3 (ab246522, 1 : 200; Abcam, USA), HIF-1*α* (ab179483, 1 : 500; Abcam, USA), and F4/80 (a monoclonal antibody specific for alveolar macrophages, ab6640, 1 : 200; Abcam, USA) overnight at 4°C in a humidifying box, followed by washing with PBS three times (5 min each), the slides were exposed to the secondary antibody Donkey anti-Rabbit IgG H&L (Alexa Fluor 594) (ab150076, 1 : 500; Abcam, USA) for 1 h at room temperature in the dark. Nuclei were visualized by staining with DAPI (P0131, Beyotime Biotechnology, Shanghai, China) for 5 min. After washing with PBS, the sections were observed with a fluorescence microscope (EVOS FL AutoLife Technologies), and representative fields were chosen for application.

### 2.9. Immunohistochemistry

The paraffin-embedded sections of mouse left lung tissues were blocked with 3% hydrogen peroxide for 15 min to inactivate endogenous peroxidases and then incubated overnight at 4°C with rabbit monoclonal anti-Drp-1 (ab184247, 1 : 1000; Abcam, USA) and rabbit polyclonal anti-Fis-1 (ab229969, 1 : 750; Abcam, USA). PBS instead of primary antibody was used as a negative control for immunofluorescence staining. The sections were washed and subsequently incubated with a secondary antibody (PV-0023-2, Bioss, China) for 1 h at room temperature. Then the sections were stained with DAB until the stain developed. Six fields of immunohistochemical images were randomly selected and semi-quantitative analysis of Drp-1 and Fis-1 was performed using the ImageJ software as previously described [34].

### 2.10. Measurement of Oxidative Stress

The Malondialdehyde (MDA) ELISA Kit (E-EL-0060c, Elabscience Biotechnology, Wuhan, China), Glutathione (GSH) ELISA Kit (E-EL-0026c, Elabscience Biotechnology, Wuhan, China), and Superoxide Dismutase (SOD_2_) ELISA Kit (CSB-EL022398MO, Wuhan, China) were used for measuring the levels of oxidative stress. The lung tissues after modeling from different groups were placed in cold saline (1: 9, w/v, one gram of tissue to 9 mL of PBS), homogenized on ice, and centrifuged at 12,000 rpm for 15 min. The supernatant from lung tissue or cell culture for detecting the levels of MDA, GSH, and SOD_2_ according to the manufacturer's instructions.

### 2.11. Measurement of ATP Content

ATP was measured using a bioluminescence assay kit (S0026, Beyotime Biotechnology, Shanghai, China). Briefly, the mouse left lung tissues or RAW264.7 cells after modeling from different groups were immediately lysed in the lysis buffer provided with the kit. The supernatant was collected by centrifugation at 12,000 rpm for 15 min at 4°C. The concentration of ATP in the samples was determined by mixing 20 *μ*L supernatant with 100 *μ*L luciferase reagent. A standard curve was prepared using a series of standards at known concentrations. The luminescence of each sample was measured on a microporous plate photometer (BioTek Instruments Inc, USA).

### 2.12. Measurement of Mitochondrial ROS (mROS)

mROS was measured using the MitoSOX™ Red mitochondrial superoxide indicator (M36008, Thermo Fisher, USA). MitoSOX is a novel fluorogenic probe for sensitive selective detection of superoxide (instead of reactive nitrogen species) in the mitochondria of live cells. Once in the mitochondria, the MitoSOX reagent is oxidized by superoxide and exhibits red fluorescence. The left lung tissues after modeling from different groups were immediately cut and digested with collagenase type I (17018029, Thermo Fisher, USA) on a shaker at 37°C for 30 min. The samples were filtered two times using a cell mesh, and then the red blood cells were lysed with red blood cell lysis buffer (R1010, Solarbio, China), and followed by centrifugation to obtain the single cells. The obtained mouse single cells or RAW264.7 cells after modeling *in vitro* were seeded into 24-well plates at a density of 5 × 10^5^ cells/mL. After cells had adhered to the wells, the cells were incubated with MitoSOX (5 *μ*m) for 10 min in the dark and nuclei were visualized by staining with Hoechst 3342 (C0030, Solarbio, China) for 20 min at room temperature as previously described [35]. The stained cells were then washed with DMEM and observed using a fluorescence microscope (EVOS FL AutoLife Technologies).

### 2.13. Mitochondrial Membrane Potential (MMP) Assay

The JC-10 assay (Solarbio, Beijing, China) was used to detect the MMP of RAW264.7 cells from different groups *in vitro* or the single-cell suspension extracted from mouse lung tissues. JC-10 is a lipophilic, cyanocyanine cationic dye that can selectively penetrate mitochondria and reversibly emit red fluorescence to green fluorescence in the case of reduced membrane potential. The healthy cells have a high membrane potential and the aggregated JC-10 exhibits red fluorescence, when the MMP was depolarized like apoptosis, the JC-10 monomers exhibited green fluorescence, and simultaneously, red fluorescence was reduced. The cells were seeded into 6-well plates at a density of 1 × 10^6^ cells/mL. After the cells had adhered, the cells were incubated with JC-10 staining working solution at 37°C in 5% CO_2_ for 20 minutes. The stained cells were then washed with dyeing buffer three times. The fluorescence intensity for both aggregated and monomeric forms of JC-10 was measured by fluorescence microscopy (EVOS FL AutoLife Technologies).

### 2.14. Western Blotting

The left lung tissues or RAW264.7 cells *in vitro* after modeling from different groups were lysed in radio immunoprecipitation assay lysis buffer on ice, and protein concentrations were measured using a Bicinchoninic acid protein assay kit (P0011, Beyotime Biotechnology, Shanghai, China). Subsequently, equal amounts of protein from each group were loaded onto 10% sodium dodecyl sulfate polyacrylamide gel electrophoresis. Proteins were then transferred onto polyvinylidene fluoride membranes and the membranes were blocked with 5% nonfat milk in TBS-Tween for 1 h at room temperature. Then, the membranes were incubated overnight at 4°C with the primary antibodies against loading control *β*-actin (4970S, 1 : 1000; Cell Signaling Technology, USA) and rabbit monoclonal anti-SIRT3 (ab246522, 1 : 1000; Abcam, USA), rabbit monoclonal anti-HIF-1*α* (ab179483, 1 : 1000; Abcam, USA), rabbit monoclonal anti-DRP-1 (ab184247, 1 : 1000; Abcam, USA), rabbit polyclonal anti-Fis-1 (ab229969, 1 : 2500; Abcam, USA), rabbit monoclonal anti-Mfn-2 (ab124773, 1 : 5000; Abcam, USA). The membranes were washed three times with TBST, and incubated for 1 h with the secondary antibody (goat anti-rabbit H&L IRDye@800 CW, ab216723,1 : 10000; Abcam, USA) at room temperature. Proteins were detected using the Alpha Innotech System (BioRad), and quantified using the ImageJ software.

### 2.15. RNA Extraction and Real-Time Quantitative PCR (RT-qPCR)

Total RNA was extracted from the left lung tissue of mice after modeling using the RNAiso Plus Kit (9109, TAKARA, Japan) following the manufacturer's instructions. After that, 1 *μ*g of total RNA was used for cDNA synthesis with the PrimeScript™ RT Reagent Kit (RR047A, TAKARA, Japan). Primers for the specific genes of interest were synthesized by Sangon Biotech (Shanghai) as follows: GAPDH, forward: 5′-TGTGTCCGTCGTGGATCTGA-3′, reverse: 5′-TTGCTGTTGAAGTCGCAGGAG-3′; SIRT3, forward: 5′-CTACATGCACGGTCTGTCGAA-3′, reverse: 5′- GCCAAAGCGAAGTCAGCCATA-3′; HIF-1*α*, forward: 5′-ACCTTCATCGGAAACTCCAAAG -3′, reverse: 5′- CTGTTAGGCTGGGAAAAGTTAGG-3′. Comparative 2^−ΔΔct^ method was used to calculate the relative quantification of the target gene, with GAPDH as an internal reference.

### 2.16. Lentivirus Infection

RAW264.7 cells in normal culture with good growth condition were infected with lentiviruses (Genechem, Shanghai, China) expressing short hairpin (sh) RNA (1 × 10^9^ TU/mL, MOI of 10 : 1) to knockdown SIRT3 (5′-GCAAGGTTCCTACTCCATA-3′) and HIF-1*α* (5′-CTGATAACGTGAACAAATA-3′) expression in the presence of polybrene (2 *μ*g/mL) according to the manufacturer's instructions. After infection for 72 h, levels of SIRT3 and HIF-1*α* were determined by western blotting, and the data were used to construct the H/R model. The cells were randomly divided into six groups: Control, H/R, H/R+shRNA (negative control for lentiviruses), H/R+S (H/R+SIRT3 shRNA, knockdown of SIRT3 expression after lentivirus infection), H/R+H (H/R+HIF-1*α* shRNA, knockdown of HIF-1*α* expression after lentivirus infection), and H/R+H+S (H/R+SIRT3 shRNA+HIF-1*α* shRNA, knockdown of SIRT3 and HIF-1*α* expression after lentivirus infection).

### 2.17. Cell Viability

The cell counting kit-8 (CCK-8, Beyotime Biotechnology, Shanghai, China) assay was used to evaluate cell viability. RAW264.7 cells after modeling from different groups were cultured in 96-well plates at a density of 2 × 10^5^ cells/mL in the presence of a complete medium. CCK-8 solution was added to each well and the plates were incubated at 37°C for 1 h. Absorbance at 450 nm was then measured using a microplate reader (BioTek Instruments Inc, USA).

### 2.18. Statistical Analysis

All the quantitative data were expressed as the mean ± standard error (SEM). Comparisons among three and more groups were analyzed using one-way analysis of variance. The statistical analysis was conducted using SPSS 25.0 (IBM, USA). A *P* value of less than 0.05 (*P* < 0.05) was considered statistically significant.

## 3. Results

### 3.1. Expression of SIRT3 Increased after Lung I/R

**Figure 1:**
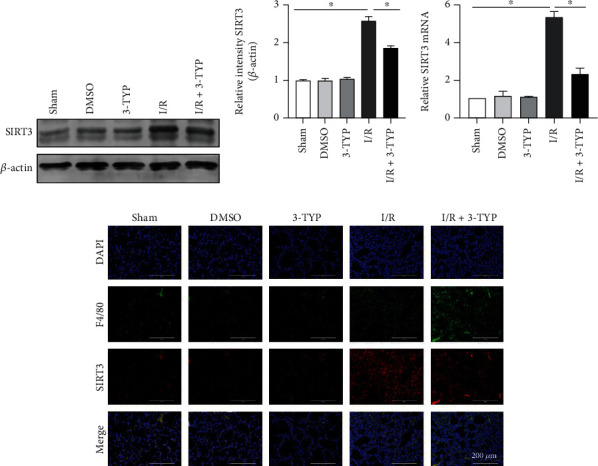
Expression of SIRT3 increased after lung I/R *in vivo*. Mice were treated with 3-TYP (50 mg/kg, i.p.) every 2 days for three times before surgery. (a) SIRT3 protein levels were detected by western blotting. (b) Protein expression of SIRT3 relative to *β*-actin. (c) Levels of SIRT3 mRNA. (d) Immunofluorescence micrographs of SIRT3 (red) and F4/80 (green) from mice of the different treatment groups. DAPI was used to stain nuclei. Scale bar: 200 *μ*m. The data are expressed as mean ± SEM (*n* = 3 for all panels), ∗*P* < 0.05.

We had previously shown that the injured lung tissue was more significantly repaired at 24 h after lung I/R compared to 6 h *in vivo*. Therefore, we chose the 24 h time point to investigate the possible role of SIRT3 in LIRI. The function of SIRT3 was blocked using 3-TYP prior to surgery. As shown in Figures 1(a)–1(c), DMSO and 3-TYP treatment exerted no significant changes for both protein and mRNA levels of SIRT3 compared with the Sham group. However, the level of SIRT3 increased significantly in the I/R group while the addition of 3-TYP to the I/R group of mice significantly inhibited the expression of SIRT3 (*P* < 0.05). This trend in SIRT3 expression was validated through immunofluorescence analysis (Figure 1(d)). In addition, the SIRT3 staining was overlaid with localization of the F4/80, suggesting that SIRT3 protein is expressed in macrophage cells.

### 3.2. Inhibition of SIRT3 Aggravated Lung Injury and Inflammation after Lung I/R

**Figure 2:**
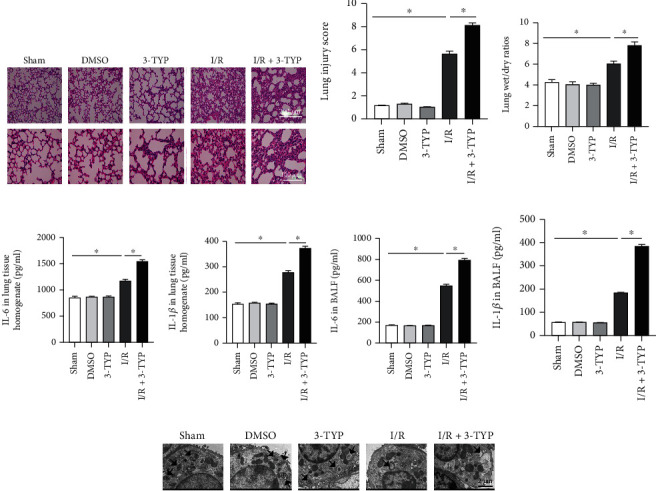
Inhibition of SIRT3 aggravated lung injury and inflammation after lung I/R *in vivo*. Histopathological injury, W/D ratios, ultrastructural changes, and inflammatory response were determined 24 h after reperfusion. (a) H&E staining of mouse lung tissue (*n* = 3 per group). Scale bar: 200 *μ*m and 100 *μ*m. (b) Pathological scores were assessed based on images in panel (a). (c) W/D ratios of lung tissues. (d) Levels of IL-6 and (e) IL-1*β* in lung tissue homogenate as measured by ELISA. (f) Levels of IL-6 and (g) IL-1*β* in BALF as measured by ELISA. The data are expressed as mean ± SEM (*n* = 6 for panels b, c, d, e, f, g), ∗*P* < 0.05. (h) Ultrastructural changes were assessed using TEM (*n* = 3 per group). Arrows indicate lamellar bodies. Scale bar, 2 *μ*m.

To investigate the effect of SIRT3 on lung I/R, the lung injury was examined at 24 h after lung I/R *in vivo*. We performed H&E staining to assess the histopathological injury. As shown in Figure 2(a), the Sham group exhibited normal lung tissue structures and no notable differences were observed for the DMSO and 3-TYP groups. In contrast, severe lung damage was observed for the I/R group, including lung edema, widespread alveolar collapse, increased alveolar thickness, and infiltration of leucocytes, and in the presence of 3-TYP, the lung injury was aggravated. The lung injury score paralleled the histological assessment where the lung injury score for the I/R +3-TYP group was higher than the I/R group (*P* < 0.05, Figure 2(b)). The W/D ratios and levels of inflammatory cytokines IL-6 and IL-1*β* were determined to evaluate lung edema and secretion of inflammatory cytokines. The W/D ratios (Figure 2(c)) and the levels of IL-6 (Figure 2(d)) and IL-1*β* (Figure 2(e)) in lung tissue homogenate as well as IL-6 (Figure 2(f)) and IL-1*β* (Figure 2(g)) in BALF were increased in the I/R group compared with the Sham group, and the levels increased more significantly after 3-TYP treatment (*P* < 0.05).

Next, we observed the ultrastructural changes using TEM and noted that the number of type II epithelial microvilli and lamellar bodies were reduced in the I/R group. More serious structural changes, including the almost total loss of type II epithelial microvilli and the absence of lamellar bodies were detected in the I/R+3-TYP group. However, these structural injuries were not seen in the other three groups (Figure 2(h)).

### 3.3. Inhibition of SIRT3 Increased Mitochondrial Oxidative Stress after Lung I/R

**Figure 3:**
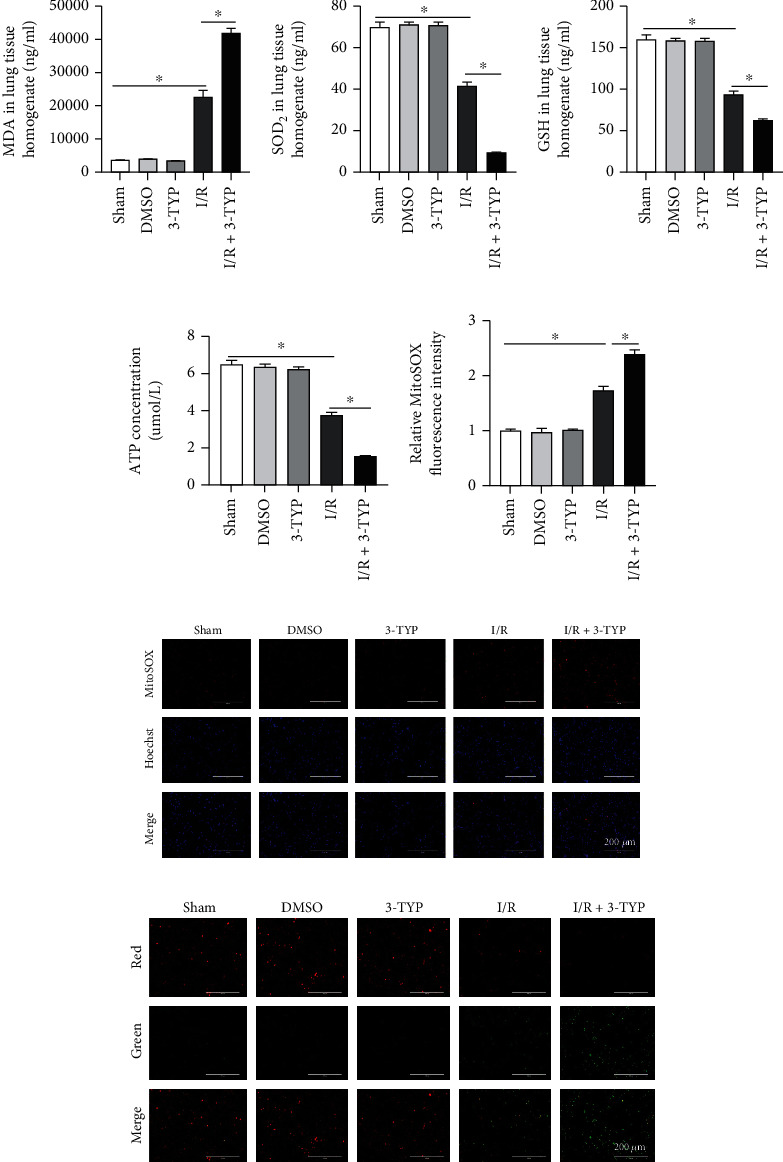
Inhibition of SIRT3 increased mitochondrial oxidative stress, but decreased ATP and MMP after lung I/R *in vivo*. (a–c) MDA, SOD_2,_ and GSH levels in lung tissues, respectively. (d) The amount of ATP. The data are expressed as mean ± SEM (*n* = 6 for panels a, b, c, d), ∗*P* < 0.05. (e, f) MitoSOX fluorescence staining and its fluorescence intensity (*n* = 3 per group). (g) Images showing the change in MMP (*n* = 3 per group).

Oxidative stress is an initial and crucial factor in the development of LIRI. The expression of MDA, SOD_2_, and GSH was determined to assess the effect of SIRT3 on I/R-induced lung oxidative stress *in vivo*. No major differences in MDA levels were noted in the DMSO and 3-TYP groups (Figure 3(a)). However, MDA levels increased in the I/R group and the highest MDA levels were observed after suppressing the function of SIRT3 (*P* < 0.05). On the other hand, SOD_2_ and GSH levels decreased in the I/R group when compared with the Sham group of mice. The lowest SOD_2_ and GSH levels were observed in the I/R+3-TYP group (Figures 3(b) and 3(c)), indicating an inhibition of oxidative stress by SIRT3. In addition, ATP content was measured and a significant reduction was noted in the I/R+3-TYP group when compared with the I/R group (Figure 3(d)).

Furthermore, MitoSOX fluorescence intensity was markedly increased in the I/R group compared with the Sham group, and no major differences were noted in the Sham, DMSO, and 3-TYP groups. MitoSOX fluorescence intensity increased in the I/R+3-TYP group compared to the I/R group (Figures 3(e) and 3(f)). The presence of MMP was detected using the JC-10 dye *in vivo*. As shown in Figure 3(g), the red fluorescence was markedly decreased in the I/R group while the green fluorescence was more pronounced compared to the Sham group, suggesting a decrease in MMP. After suppressing the function of SIRT3, MMP decreased compared to the I/R group. The results demonstrated that inhibition of SIRT3 can induce mitochondrial dysfunction and increases oxidative stress after lung I/R.

### 3.4. Inhibition of SIRT3 Increased Mitochondrial Fission after Lung I/R

**Figure 4:**
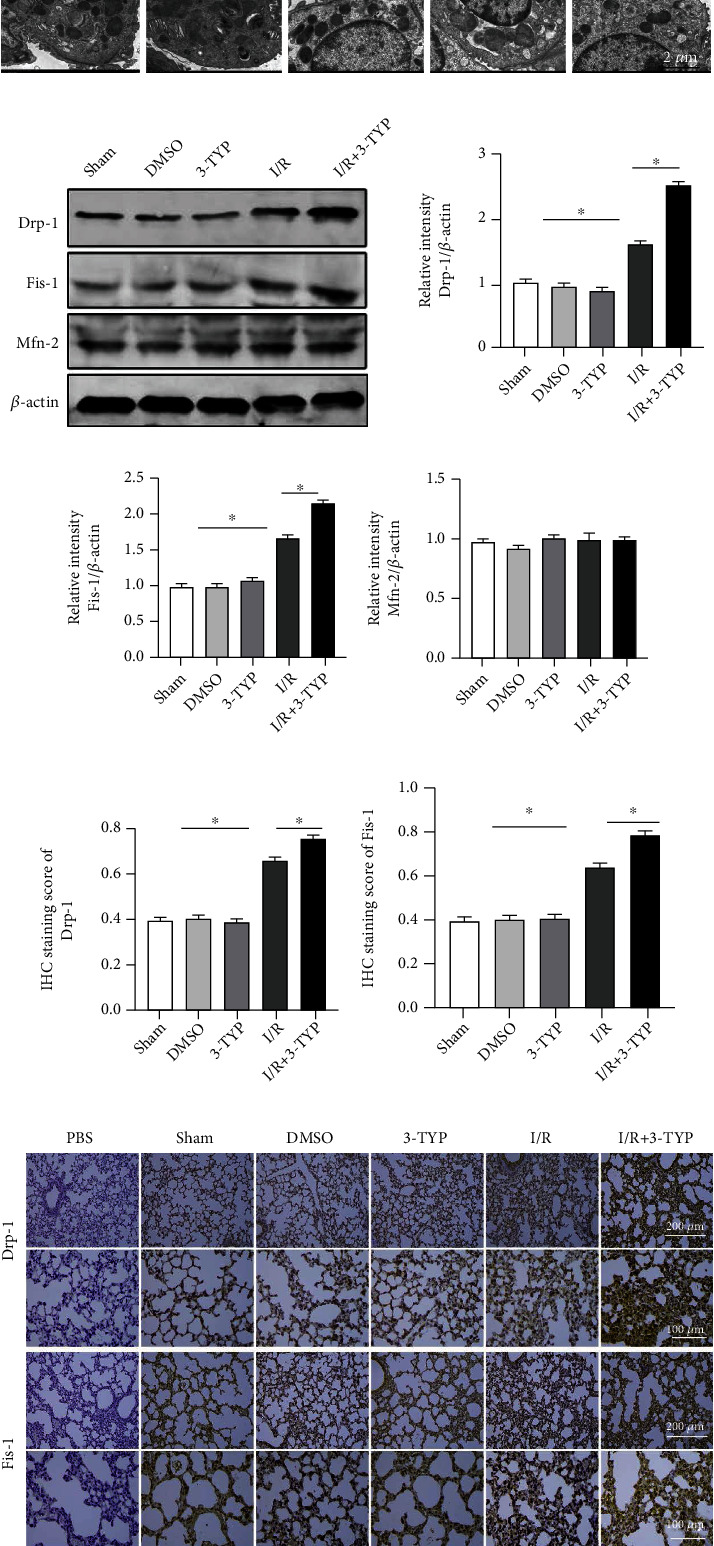
Inhibition of SIRT3 decreased the number of mitochondria, increased the levels of Drp-1 and Fis-1, and led to excessive mitochondrial fission after I/R *in vivo*. (a) Mitochondrial changes were assessed using TEM. Scale bar, 2 *μ*m. (b) Drp-1, Fis-1, and Mfn-2 protein levels detected by western blotting. (c–e) Drp-1, Fis-1, and Mfn-2 protein expression relative to *β*-actin. (f–h) Lung Drp-1 and Fis-1 protein expression detected by immunohistochemistry and semiquantitative analysis of expression in representative lung sections. Scale bar: 200 *μ*m and 100 *μ*m. The data are expressed as mean ± SEM (*n* = 3 for all panels), ∗*P* < 0.05.

To investigate the relationship between SIRT3 and mitochondria in LIRI, TEM was conducted to observe the lung mitochondrial changes in I/R regions. In the I/R group, we observed that the mitochondria in macrophages were swollen and numbers were markedly decreased when compared to the Sham group. Furthermore, when compared with the I/R group, the 3-TYP pretreatment increased mitochondrial fission with fewer mitochondria present after 24 h of reperfusion *in vivo* (Figure 4(a)). We performed western blotting to detect changes in amounts of Drp-1 and Fis-1 proteins and found that Drp-1 and Fis-1 were expressed more significantly in the I/R group compared to the Sham group (*P* < 0.05). Moreover, when the function of SIRT3 was suppressed, expression of Drp-1 and Fis-1 was higher than the I/R group. However, the mitochondrial fusion-related protein, Mfn-2, was not significantly changed in all groups (Figures 4(b)–4(e)). A similar trend was observed with immunohistochemistry whereby 3-TYP pretreatment increased the expression of Drp-1 and Fis-1 (Figures 4(f)–4(h)). These data indicated that inhibition of SIRT3 leads to excessive mitochondrial fission after lung I/R.

### 3.5. Inhibition of SIRT3 Increased the Expression of HIF-1*α* after Lung I/R

**Figure 5:**
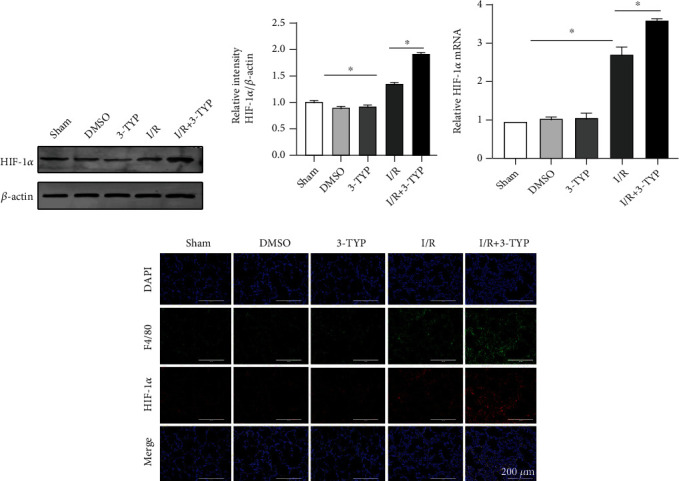
Inhibition of SIRT3 increased the expression of HIF-1*α* after lung I/R *in vivo*. (a) HIF-1*α* protein levels by western blotting. (b) Expression of HIF-1*α* protein relative to *β*-actin. (c) Levels of HIF-1*α* mRNA. (d) Immunofluorescence micrographs of HIF-1*α* (red) and F4/80 (green) in lung tissues. DAPI was used to stain nuclei. Scale bar: 200 *μ*m. The data are expressed as mean ± SEM (*n* = 3 for all panels), ∗*P* < 0.05.

Expression of HIF-1*α* protein was markedly increased after 24 h of reperfusion *in vivo* and no major differences were noted in the Sham, DMSO, and 3-TYP groups (Figures 5(a) and 5(b)). Furthermore, when 3-TYP was administered to suppress the function of SIRT3, levels of HIF-1*α* protein increased significantly compared to the I/R group (*P* < 0.05). The increase in expression of HIF-1*α* was also observed at the mRNA level (Figure 5(c)). Western blotting and RT-qPCR observations were confirmed by immunofluorescence which also showed that HIF-1*α* protein levels were markedly increased after I/R injury and pretreatment with 3-TYP relative to the I/R group (Figure 5(d)). In addition, HIF-1*α* protein was expressed in macrophages. These findings suggest that inhibition of SIRT3 significantly increased the level of HIF-1*α*.

### 3.6. Knockdown SIRT3 Increased Mitochondrial Oxidative Stress by Regulating the Expression of HIF-1*α* In Vitro

**Figure 6:**
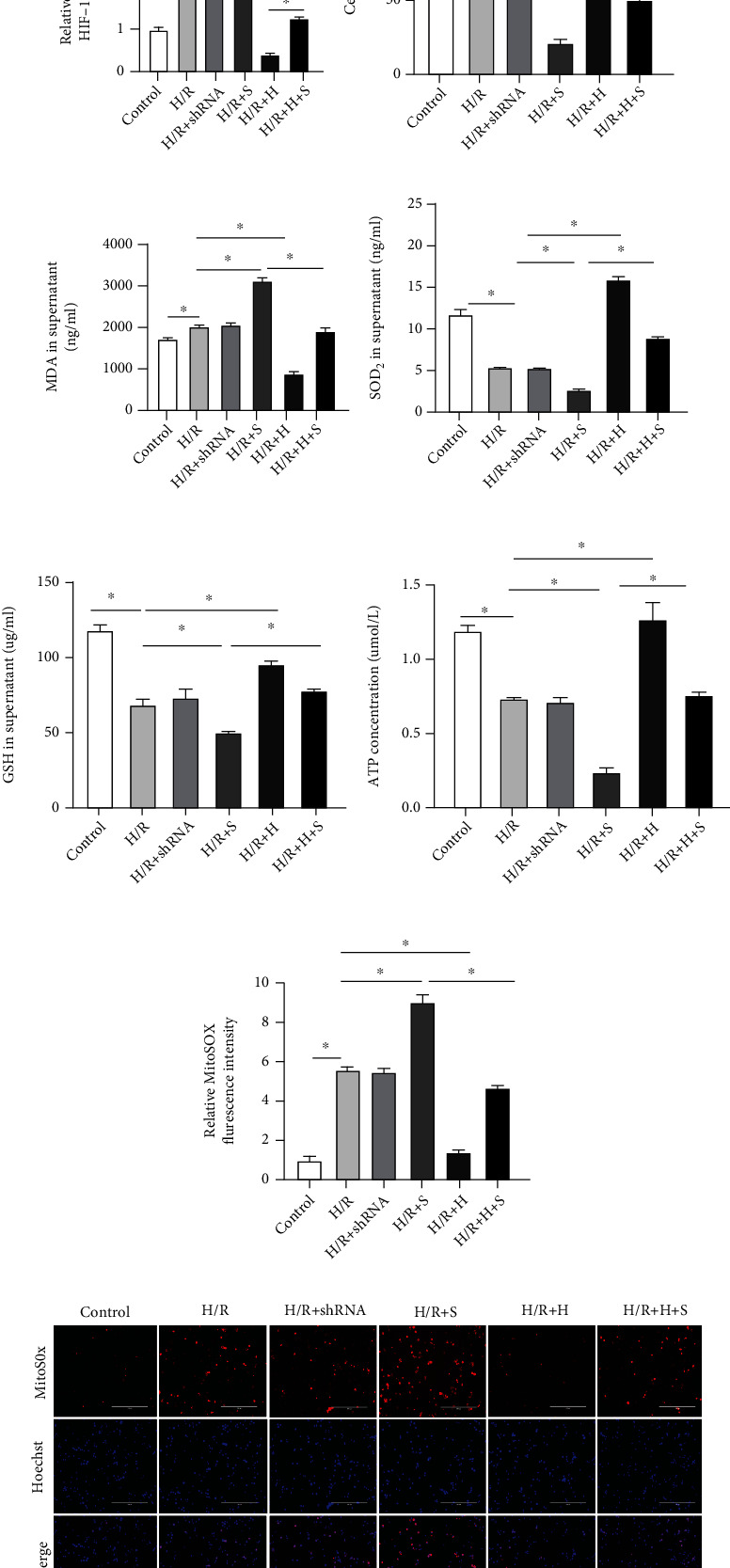
Knockdown SIRT3 increased mitochondrial oxidative stress, decreased ATP content, and MMP by regulating HIF-1*α* after H/R *in vitro*. A lentiviral vector expressing shRNA targeting SIRT3 and HIF-1*α* individually was used to knockdown the expression of SIRT3 and HIF-1*α*. Cells were randomly divided into six groups: Control, H/R, H/R+shRNA, H/R+S (H/R+SIRT3 shRNA), H/R+H (H/R+HIF-1*α* shRNA), and H/R+H+S (H/R+SIRT3 shRNA+HIF-1*α* shRNA) groups. Cells and supernatants from each group were collected after 24 h reoxygenation. (a) SIRT3 and HIF-1*α* protein levels were detected by western blotting. (b, c) Expression of SIRT3 and HIF-1*α* protein relative to *β*-actin. (d) Cell viability of RAW264.7 macrophage cells determined by the CCK-8 kit. (e–g) MDA, SOD_2_, and GSH levels in the supernatant. (h) ATP content. (i, j) MitoSOX fluorescence staining and fluorescence intensity. (k) Images showing the change in MMP. The data are expressed as mean ± SEM (*n* = 3 for all panels), ∗*P* < 0.05.

From the previous experiments described previously, we noted that SIRT3 and HIF-1*α* were detectable in macrophages. Hence, we performed *in vitro* studies using RAW264.7 murine macrophage cells to investigate whether SIRT3 protects mitochondrial function by regulating the expression of HIF-1*α*. First, cells were infected with a lentiviral vector expressing shRNA targeting SIRT3 and HIF-1*α*, individually. We performed western blotting to confirm the knockdown effect and noted that SIRT3 and HIF-1*α* expression were significantly decreased by the SIRT3 and HIF-1*α* shRNAs, respectively. Moreover, consistent with observations from the *in vivo* experiments, expression of SIRT3 and HIF-1*α* proteins increased after H/R. After knocking down SIRT3, HIF-1*α* expression increased significantly (*P* < 0.05). However, SIRT3 expression levels did not change significantly after knocking down HIF-1*α* (Figures 6(a)–6(c)). The results suggest that SIRT3 could possibly act as an upstream regulator of HIF-1*α* expression.

Cell viability of the lentivirus infected cells was then tested and we noted H/R had reduced cell viability (Figure 6(d)) when compared with the Control group. Knockdown SIRT3 led to significantly lower levels of cell viability relative to the H/R group, which was partially reversed by knocking down HIF-1*α* (*P* < 0.05). Consistent with the *in vivo* results, SIRT3 knockdown increased MDA levels while levels of SOD_2_ and GSH were decreased *in vitro* when compared to the H/R group, all of which were reversed when HIF-1*α* was knocked down (*P* < 0.05, Figures 6(e)–6(g)). A similar trend in SOD_2_ levels was observed for the ATP content, knockdown SIRT3 decreased the ATP content when compared with the H/R group (Figure 6(h)). MitoSOX fluorescence intensity was significantly higher in the H/R group than the Control group, while in the H/R+H group, mROS levels were markedly decreased. On the other hand, when SIRT3 was knocked down, mROS levels were higher than the H/R group, which was partially reversed by knocking down HIF-1*α* (Figures 5(i) and 5(j)). As expected, MMP was markedly decreased after SIRT3 knocking down compared to the H/R group, while knocking down HIF-1*α* mitigated mitochondrial injury (Figure 6(k)). This suggests that knockdown SIRT3 could increase mitochondrial oxidative stress and cause mitochondrial dysfunction by increasing the expression of HIF-1*α*. Taken together, the SIRT3-HIF-1*α* pathway may be a good therapeutic target.

### 3.7. Knockdown SIRT3 Increased the Levels of Drp-1 and Fis-1 by Regulating the Expression of HIF-1*α* In Vitro

**Figure 7:**
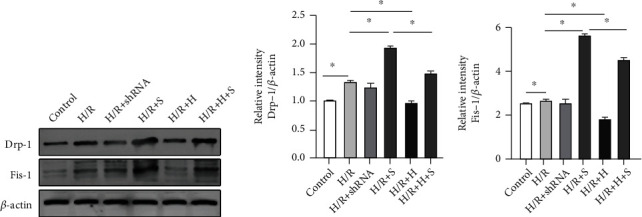
Knockdown SIRT3 increased the levels of Drp-1 and Fis-1 by regulating HIF-1*α* after H/R *in vitro*. (a) Drp-1 and Fis-1 protein levels determined by western blotting. (b, c) Expression of Drp-1 and Fis-1 proteins relative to *β*-actin. The data are expressed as mean ± SEM (*n* = 3 for all panels), ∗*P* < 0.05.

To investigate whether SIRT3 participates in mitochondrial fission by regulating HIF-1*α*, expressions of Drp-1 and Fis-1 were detected by western blotting. We noted that Drp-1 and Fis-1 protein levels were significantly increased in the H/R group compared to the Control group. Furthermore, HIF-1*α* knockdown decreased Drp-1 and Fis-1 expression relative to the H/R group (*P* < 0.05). However, for the SIRT3 knockdown, expressions of Drp-1 and Fis-1 were higher than the H/R group. On the other hand, expression of both proteins was notably reduced for the HIF-1*α* knockdown (Figures 7(a)–7(c)).

### 3.8. Inhibition of Drp-1 Alleviated Mitochondrial Oxidative Stress In Vitro

**Figure 8:**
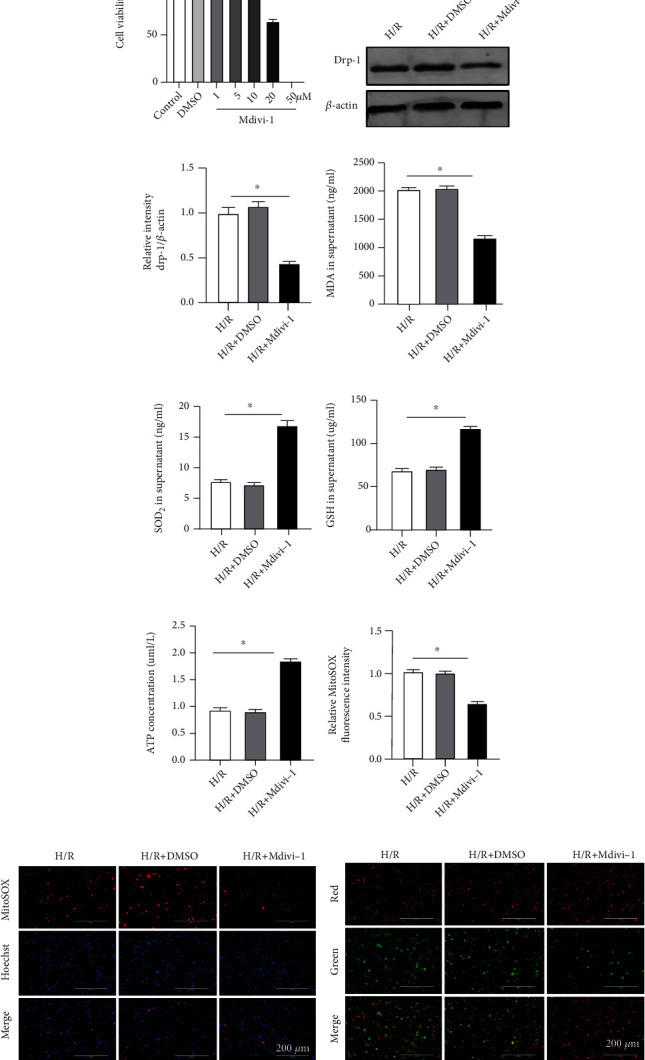
Inhibition of Drp-1 reduced mitochondrial fission, decreased mitochondrial oxidative stress, increased ATP content and MMP after H/R *in vitro*. RAW264.7 cells were subjected to Mdivi-1 treatment (10 *μ*m) for 1 h before induction of hypoxia. (a) Cell viability of RAW264.7 cells after treatment with different concentrations of Mdivi-1 measured with the CCK-8 kit. (b) Western blotting of Drp-1 protein. (c) Expression of Drp-1 protein relative to *β*-actin. (d–f) MDA, SOD_2_, and GSH levels in the supernatant of RAW264.7 cells. (g) The content of ATP. (h, i) MitoSOX fluorescence staining and fluorescence intensity. (j) Representative images showing the change in MMP. The data are expressed as mean ± SEM (*n* = 3 for all panels), ∗*P* < 0.05.

To explore the potential relationship between mitochondrial fission and oxidative stress and the specific regulatory mechanism involved, we used Mdivi-1 (Drp-1 selective inhibitor, dissolved in DMSO, Sigma-Aldrich, USA) to inhibit the function of Drp-1 [15]. RAW264.7 cells were treated with different concentrations (0–50 *μ*m) of Mdivi-1 for 1 h as previously described [36] and cell viability was measured using the CCK-8 kit. As shown in Figure 8(a), Mdivi-1 at ≥20 *μ*m showed significant inhibition of cell viability compared to the Control group (*P* < 0.05), and no major differences were noted for the other concentrations tested. Hence, for the rest of the experiments, cells were treated with 10 *μ*m of Mdivi-1 for 1 h followed by exposure to hypoxic conditions *in vitro*. We performed western blotting to verify the efficiency of Mdivi-1 inhibitory properties and observed that expression of Drp-1 was significantly decreased in the H/R+Mdivi-1 group when compared with the H/R group while DMSO did not affect Drp-1 expression (Figures 8(b)–8(c)). After confirming that the function of Drp-1 was suppressed, we examined the indicators of mitochondrial oxidative stress. In the absence of functional Drp-1, MDA levels decreased while levels of SOD_2_ and GSH increased (*P* < 0.05) (Figures 8(d)–8(f)). As shown in Figure 8(g), ATP content increased in the presence of Mdivi-1 compared to the H/R group while mROS decreased (Figures 8(h)–8(i)). MMP also exhibited a similar profile in the H/R+Mdivi-1 group (Figure 8(j)), with the red fluorescence intensity markedly increased while the intensity of green fluorescence decreased. The results demonstrated that excessive mitochondrial fission could increase oxidative stress and lead to mitochondrial dysfunction.

## 4. Discussion

Lung transplantation, hemorrhagic shock and pulmonary embolism can lead to the occurrence of LIRI which is the main cause of pulmonary edema and respiratory failure. A previous study had reported that melatonin ameliorates myocardial I/R injury by activating SIRT3 which regulates oxidative stress and apoptosis [37]. A different study also showed that SIRT3 reduces inflammation and mitigated endotoxin-induced acute lung injury (ALI) [38]. In the present study, we showed that inhibition of SIRT3 aggravates LIRI in mice. This may explain SIRT3's ability to inhibit mitochondrial fission, reduce oxidative stress and increase MMP by regulating HIF-1*α*. The SIRT3-HIF-1*α* signaling pathway may serve as an important therapeutic target for alleviating LIRI.

In normal postmitotic cells, mitochondria produce over 90% of cellular energy in the form of ATP as well as other important intermediates such as NADH and NADPH, and mitochondria are also the major site for production of ROS [39]. Oxidative stress occurs when the continuous generation of ROS leads to an imbalance between the production of oxygen radicals and antioxidant potential resulting in DNA, protein, and lipid damage, which occurs in many human diseases such as diabetes, atherosclerosis, and ALI [40, 41]. An earlier study had shown that SIRT3 depletion during hypertension increases vascular oxidative stress, promotes endothelial dysfunction, vascular inflammation, and end-organ damage [42]. Additionally, decreased levels of SIRT3 *in vivo* and *in vitro* renal I/R and overexpression of SIRT3 modulated oxidative injury, repressed inflammatory damage, and reduced tubular epithelial cell apoptosis [43]. Sufficient amounts of SIRT3 may be important to regulate oxidative stress during I/R. In the current study, we determined that the levels of SIRT3 increased at 24 h after lung I/R *in vivo*. Given the protective effects of SIRT3 in a renal I/R model, the increase may be an endogenous protective mechanism for I/R injury. Interestingly, recent studies found that SIRT3 consumption is associated with many factors such as sedentary lifestyle, smoking, aging, metabolic status, and inflammation [44, 45]. The increased release of SIRT3 under these conditions may be to improve the body's response to harmful or pathological conditions in order to overcome abnormal metabolic, inflammatory, and oxidative stress responses. Therefore, we showed that inhibition of SIRT3 increased MDA levels but decreased the levels of SOD_2_ and GSH as well as ATP content *in vivo*. Moreover, the production of mROS was also much higher than the Sham group, leading to reduced MMP and ultimately mitochondrial dysfunction. These results are consistent with our initial hypothesis that SIRT3 participates in the regulation of oxidative stress after lung I/R.

The dynamic balance between mitochondrial fusion and fission is required to meet the biological energy requirements of cells [46]. The fusion process helps to homogenize the contents of damaged mitochondria, leading to mitochondrial elongation. Fission, on the other hand, causes mitochondrial fragmentation and promotes the clearance of damaged mitochondria through a form of selective autophagy-mitophagy [47]. Mitophagy is a selective form of microautophagy in which mitochondria are the preferred targets for autophagolysosomal degradation [48]. In diseased states or in response to injury, mitochondria undergo fragmentation into small dysfunctional units that produce excessive ROS leading to adverse cellular effects [19, 49]. Mitophagy is necessary for the clearance of dysfunctional mitochondria, and thus serves an important housekeeping role in maintaining mitochondrial function and limiting the production of damaging ROS. Excessive fission has been shown to contribute to cardiac injury under certain conditions such as pressure-overload, ischemia, and doxorubicin treatment [50, 51]. A previous study also confirmed that excessive mitochondrial fission is an important mediator of hyperoxia-induced pulmonary vascular injury [52]. Intermittent hypobaric hypoxia-induced preconditioning in rats was reported to assist the lung to adapt to severe hypoxic conditions by stabilizing mitochondrial function [53]. In another study, cigarette smoke was shown to promote mitochondrial fission and reduced fusion which led to mitochondrial oxidative stress and impaired mitochondrial respiration [54]. Our study showed that inhibition of SIRT3 significantly increased the expression of Drp-1 and Fis-1 after lung I/R *in vivo*. SIRT3 may reduce oxidative stress and protect mitochondrial function by inhibiting mitochondrial fission, thus reducing lung injury and the inflammatory response.

Hypoxia results in transcriptional upregulation of a range of genes involved in cell proliferation and survival. HIF-1*α* is a key component of the eukaryotic oxygen response system [55]. Under hypoxic conditions, degradation of HIF-1*α* is reduced, this promotes transcription of numerous genes involved in the response of cells towards hypoxia [56]. A previous study revealed that HIF-1*α* induces polarization of macrophage M1 subtypes by increasing ROS production [57]. Another study showed that SIRT3 represses tumor growth by downregulating HIF-1*α* and reducing ROS accumulation [26]. Our *in vitro* study demonstrated that knockdown SIRT3 significantly increased the expression of HIF-1*α*, promoted mROS, reduced MMP, and increased mitochondrial fission, thus contributing to mitochondrial dysfunction after reoxygenation. When HIF-1*α* was knocked down, the observed changes were reversed and cell viability increased. Hence, SIRT3 might quench oxidative stress and mitochondrial fission by decreasing HIF-1*α* expression. HIF-1*α* is related to ROS production and accumulation and also has an association with mitochondria because HIF-1*α* and ROS are both related to oxygen metabolism. One study suggested that HIF-1*α* exerts a protective role in diabetic nephropathy by upregulation heme oxygenase-1 (HO-1) expression to regulate the mitochondrial dynamics [58]. HO-1 promotes mitochondrial fusion through the increased expression of Mfn-1 and Mfn-2 and inhibits mitochondrial fission by inhibiting the mitochondrial fission mediators Drp-1 and Fis-1. As such, overexpression of HO-1 maintains mitochondrial homeostasis. Jia Shi et al. also demonstrated that dexmedetomidine ameliorates endotoxin-induced ALI *in vivo* and *in vitro* by maintaining mitochondrial dynamic homeostasis through the HIF-1*α*/HO-1 signaling pathway [59]. However, another study confirmed that hypoxia promotes mitochondrial fission through HIF-1*α* and regulates mitophagy by controlling BCL2-interacting protein 3 translocation to mitochondria in nucleus pulposus cells [60]. Inhibition of mitochondrial fission may have broad therapeutic benefits in the progression of various diseases. In the future, we propose to extend our studies to elucidate how HIF-1*α* inhibits mitochondrial fission and its regulatory mechanisms in LIRI as a means to alleviate lung injury.

We also explored the relationship between oxidative stress and mitochondrial fission. Oxidative stress can lead to endothelial and epithelial barrier dysfunction, while excess ROS upregulated proinflammatory factors and adhesion molecules which aggravated tissue damage and pulmonary edema in acute respiratory distress syndrome [61]. Scavenging ROS and alleviating oxidative stress are crucial for relieving LIRI. A recent study demonstrated that inhibition of thrombospondin-1 might prevent Drp-1 mediated mitochondrial fission and improve cardiac diastolic function by reducing ROS mediated cardiomyocyte damage in the older population [62]. Concomitantly, inhibiting mitochondrial fission with Mdivi-1 ameliorated ALI [36]. Our study also found that inhibition of Drp-1 significantly decreased the levels of oxidative stress indices such as mROS and MDA but increased SOD_2_ and GSH levels after reoxygenation *in vitro*. Furthermore, we found that inhibition of Drp-1 could improve MMP and alleviate mitochondrial dysfunction. SIRT3 may reduce oxidative stress by inhibiting mitochondrial fission and play a protective role in the lungs of LIRI patients.

While this study has demonstrated that inhibition of SIRT3 aggravates LIRI by increasing mitochondrial fission and oxidative stress, there are some limitations within the study. Firstly, our study was mainly focused on confirming that SIRT3 knockdown under H/R conditions would lead to excessive mitochondrial fission. However, determining whether Mdivi-1 treatment in the setting of SIRT3 knockdown would prevent the decline in cell viability requires a more in-depth study. Secondly, while we have shown that SIRT3 regulated mitochondrial fission through HIF-1*α*, it is not known if HIF-1*α* can directly regulate Drp-1 and Fis-1 and if so, what is the regulatory mechanism involved. Furthermore, it would be interesting to investigate if the use of agonists can alleviate the exacerbation of lung injury and inflammatory response after inhibiting SIRT3.

## 5. Conclusions

In this study, we demonstrated that downregulation of SIRT3 increases mitochondrial fission and oxidative stress by increasing the expression of HIF-1*α*, thereby aggravating LIRI. Our findings propose that the SIRT3-HIF-1*α* pathway may serve as a target for treatment of LIRI.

## Data Availability

The data used to support the findings of this study are available from the corresponding author upon request.
